# Suicide attempt using pure methanol with hospitalization of the patient soon after ingestion: case report

**DOI:** 10.1590/S1516-31802009000200011

**Published:** 2009-07-06

**Authors:** Fábio Bucaretchi, Eduardo Mello De Capitani, Paulo Roberto de Madureira, Danielle Menezes Cesconetto, Rafael Lanaro, Ronan José Vieira

**Affiliations:** 1 MD, PhD. Assistant professor, Department of Pediatrics, Poison Control Center (Centro de Controle de Intoxicações, CCI), School of Medical Sciences, Hospital das Clínicas, Universidade Estadual de Campinas (Unicamp), Campinas, São Paulo, Brazil.; 2 MD, PhD. Assistant professor, Department of Internal Medicine, Poison Control Center (Centro de Controle de Intoxicações, CCI), School of Medical Sciences, Hospital das Clínicas, Universidade Estadual de Campinas (Unicamp), Campinas, São Paulo, Brazil.; 3 MD, PhD. Assistant professor, Department of Preventive and Social Medicine, Poison Control Center (Centro de Controle de Intoxicações, CCI), School of Medical Sciences, Hospital das Clínicas, Universidade Estadual de Campinas (Unicamp), Campinas, São Paulo, Brazil.; 4 Medical student, Poison Control Center (Centro de Controle de Intoxicações, CCI), School of Medical Sciences, Hospital das Clínicas, Universidade Estadual de Campinas (Unicamp), Campinas, São Paulo, Brazil.; 5 MSc. Pharmacist, Toxicology Laboratory, Poison Control Center (Centro de Controle de Intoxicações, CCI), School of Medical Sciences, Hospital das Clínicas, Universidade Estadual de Campinas (Unicamp), Campinas, São Paulo, Brazil.

**Keywords:** Methanol, Suicide, attempted, Poisoning, Ethanol, Hemodialysis, Metanol, Tentativa de suicídio, Intoxicação, Etanol, Hemodiálise

## Abstract

**CONTEXT:**

Most patients with methanol poisoning typically show up one to several days after ingestion, presenting severe acidosis, visual disorders, or both. Reports of hospitalization less than 6 h after exposure are unusual. We describe a case of attempted suicide using methanol admitted 3 h after ingestion.

**CASE REPORT:**

A 52-year-old male was hospitalized 3 h after intentional ingestion of 150 ml of 99.9% methanol with no co-ingestion of ethanol. He was alert and cooperative, presenting nausea and vertigo, and reporting six episodes of vomiting. Physical examination showed no remarkable features. A blood sample for methanol and ethanol determination was obtained 4 h after ingestion. The result (available 10 h after ingestion) showed 70 mg/dl of methanol, without detectable ethanol. He was treated with a loading dose of 10% ethanol solution (7 ml/kg, intravenously), followed by a maintenance dose of 0.9-1.0 ml/kg/h intravenously (10 to 51 h); hemodialysis (19 to 27 h, together with 2.1 ml/kg/h of 10% ethanol intravenously); and folinic acid intravenously (50 mg every 6 h, from 4 to 51 h). He developed mild/moderate metabolic acidosis without acidemia and was discharged on day four after ophthalmological evaluation and cerebral computed tomography scan, without abnormalities. Follow-up revealed no sequelae.

**CONCLUSION:**

This could be classified as a potentially severe case of methanol poisoning, according to the amount and concentration of methanol ingested, and blood methanol concentration at 4 h. The good outcome was attributable to early hospitalization and early antidotal therapy with hemodialysis, starting at 10 and 19 h, respectively.

## RESUMO

**CONTEXTO:**

A maioria dos pacientes intoxicados por metanol se apresenta um a vários dias após a ingestão, com acidose grave e/ou alterações visuais, sendo rara a admissão com menos de seis horas da exposição. Descrevemos uma tentativa de suicídio com metanol puro admitido três horas após a ingestão.

**RELATO DE CASO:**

Homem de 52 anos, admitido três horas após ingestão intencional de 150 ml de metanol 99,9% sem co-ingestão de etanol. Ele estava alerta e cooperativo, apresentando náuseas, vertigem e relatando seis episódios de vômitos. Sem achados relevantes no exame físico. Foi coletada amostra sanguínea para determinação dos níveis séricos de metanol e etanol em quatro horas, com resultado liberado em 10 horas, mostrando metanol = 70 mg/dl e etanol não detectável. O paciente foi tratado com uma dose de ataque intravenosa (IV) de etanol 10% de 7 ml/kg, seguida por uma dose de manutenção de 0,9-1,0 ml/kg/h IV de 10 a 51 horas; hemodiálise (19 a 27 horas), recebendo, nesse período, 2,1 ml/kg/h de etanol 10% IV e oito doses de ácido folínico IV (50 mg cada 6 horas, de 4 a 51 horas). Desenvolveu acidose metabólica leve/moderada, sem acidemia, sendo liberado no quarto dia de internação após avaliação oftalmológica e realização de tomografia computadorizada cerebral, sem alterações. Acompanhamento não revelou sequelas.

**CONCLUSÃO:**

O presente caso pode ser classificado como uma intoxicação potencialmente grave por metanol, considerando a quantidade e a concentração ingerida e o nível sérico de metanol obtido em quatro horas. A boa evolução pode ser atribuída ao intervalo entre a exposição e a admissão hospitalar, e o tratamento específico com o antídoto e a hemodiálise, respectivamente iniciados em 10 e 19 horas.

## INTRODUCTION

Methanol is available, particularly in Northern Hemisphere countries, as a component of many household products (antifreeze, paint removers, windshield washer fluids, solvents and cleaners).^1^ Although suicide attempts using methanol are not uncommon, mass epidemic methanol poisonings have historically been described after ingestion of contaminated beverages, especially in countries with high rates of alcohol consumption.^1^^,^^2^ Most poisoned patients typically show up one to several days after ingestion, with severe acidosis, visual disorders, or both.^1^^,^^2^ Pancreatic lesions or permanent brain damage, particularly bilateral putamen necrosis, have also been described after severe exposure.^1^ Reports of patients admitted to hospital less than 6 h after acute methanol exposure, with subsequent determination of their acid-base status and blood methanol levels are very unusual.^3^

## CASE REPORT

A 52-year-old male weighing 70 kg who was a teetotaler and a biology researcher was admitted to hospital 3 h after intentional ingestion of 150 ml of 99.9% methanol. This is an analytical reference material stored in graduated glass laboratory apparatus, which was used for self-harm purposes by this patient. He was brought in by his wife, who had found him early in the morning vomiting, soon after the exposure. He was alert, cooperative, presenting nausea and vertigo, and reporting six episodes of vomiting, without other complaints. He said he had not co-ingested any ethanol. He had been taking bupropion, clonazepam and oxycarbazepine under irregular psychiatric treatment for depression, for the preceding four months.

Physical examination showed no remarkable features. A blood sample for methanol and ethanol determination (gas chromatography) was obtained 4 h after ingestion, and the result came out 6 h later (10 h after ingestion), showing 70 mg/dl of methanol and no detectable ethanol. With this result, it was decided to institute a loading dose of 10% ethanol solution (7 ml/kg intravenously), followed by a maintenance dose of 0.9-1.0 ml/kg/h intravenously, for 42 h, and hemodialysis was also indicated.^1^ An eight-hour course of hemodialysis was then started 19 h after ingestion, during which 2.1 ml/kg/h of 10% ethanol was administered.^1^ Eight intravenous doses of folinic acid (50 mg in 5% dextrose, for 30-60 minutes every 6 h) were also given over a 48-hour period, starting 4 h after ingestion.^1^

**Figure 1.**
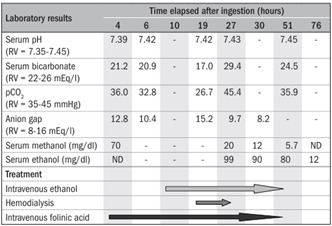
Main laboratory results and treatment details according to time elapsed after ingestion.

Figure 1 summarizes the main laboratory results and treatment measures according to the time after ingestion. Blood glucose, complete blood counts, electrolytes, amylase, liver function tests, lactate dehydrogenase, creatinine, urea nitrogen and electrocardiographic analyses were within reference values.

The patient was discharged on day 4, after undergoing ophthalmological evaluation and a cerebral computed tomography scan, which did not show any abnormalities. Follow-up did not show any sequelae, and the patient is currently under regular psychiatric treatment, with good compliance so far.

RV = reference values; ND = not detected.

## DISCUSSION

Methanol poisoning and methanol level interpretation imply consideration of certain variables like time since ingestion, concentration and dose of methanol, co-ingestion of ethanol and acid-base status.^1^^,^^2^^,^^3^^,^^4^ Methanol is rapidly absorbed, and peak values are reached within 30- 60 minutes. The kinetics follows a zero-order model with a rate of 8.5 mg/dl/h.^1^^,^^3^ Considering the total amount of methanol ingested by this patient (150 ml) and his weight, the estimated peak blood level could be calculated as 282 mg/dl.^1^^,^^3^ However, considering the several episodes of vomiting, the elimination rate and the methanol blood level 4 h after ingestion (70 mg/dl), it might be inferred that probably only one third of the ingested dose (around 50 ml) was actually absorbed.

A recent systematic review showed that median blood methanol levels were inversely correlated with the delay in seeking medical care.^3^ It was shown that, among 173 poisoned patients who fulfilled the inclusion criteria, only 18 (10.4%) had been admitted to hospital less than 4 h after methanol ingestion (median level of 212 mg/dl, ranging from 10 mg/dl to 570 mg/dl), and 13 became acidotic (serum bicarbonate ranging from 5 to 21 mEq/l; median of 14 mEq/l).^3^ Our case showed progressive mild/moderate metabolic acidosis (4 to 19 h after ingestion), without acidemia. No low bicarbonate levels were observed during or after hemodialysis.

In general, blood peak methanol concentrations above 50 mg/dl may indicate serious poisoning, particularly if anion gap metabolic acidosis is present.^1^ An inverse correlation between initial arterial pH values and plasma formic acid concentration has been shown in methanol poisoning patients.^3^ Although controversial, antidotal treatment and hemodialysis have been routinely recommended for patients admitted with methanol blood levels > 20 mg/dl and > 50 mg/dl, respectively.^1^^-^^2^ In addition, some authors have suggested that a delay of 12 h to 24 h may be needed in methanol poisoning cases before overt acidosis is manifested.^1^ Following these suggestions, we decided to treat our patient with intravenous ethanol, hemodialysis and intravenous folinic acid.^1^

If administered soon after methanol exposure, ethanol and fomepizole should prevent or reduce the formation of toxic metabolites.^1^ Ethanol has approximately 10 times greater affinity for alcohol dehydrogenase than shown by methanol, and has been used as an antidote since the 1940s.^1^ The ethanol doses used were based on average pharmacokinetic values, achieving serum ethanol concentrations from 80 mg/dl to 99 mg/dl, i.e. close to the target concentration (100 mg/dl).^1^ Although the efficacy of intravenous fomepizole has already been established in methanol poisoning cases,^4^ this antidote is still quite expensive and is not available in Brazil.

Hemodialysis has been used routinely to correct acidosis, remove methanol and toxic metabolites, and shorten the duration of hospitalization.^1^^-^^2^ Although the traditional indication for hemodialysis is a serum concentration of methanol higher than 50 mg/dl, the prognosis seems mainly related to the degree of acidosis and not to the blood methanol concentration.^1^ Since the morbidity and mortality associated with methanol poisoning have been attributed to formic acid accumulation, adjuvant therapy with folinic acid might be beneficial. Folate enhances formic acid metabolization, and it has been postulated that it reduces the toxicity.^1^

Liu et al.^5^ analyzed 43 methanol-related deaths in Ontario, Canada, of which 10 (23.2%) had been caused by ingestion of pure methanol, mostly intentional (7/10 of these cases), and pointed out the potential severity of pure methanol exposure.^5^ However, these authors did not provide any details regarding the amount ingested, the time elapsed from ingestion to hospital admission and the treatment used. Therefore, direct comparison with our case is not possible.

We also performed a systematic search in the following electronic databases: Medical Literature Analysis and Retrieval System Online (Medline) through PubMed, EMBASE, Cochrane Library and LILACS. The search strategies used and the results are shown in Table 1. The results using the MeSH and DeCS terms showed that there were few papers relating to cases of poisoning by pure methanol (analytical standard). Most of the available articles described the clinical effects and laboratory analysis results from cases of accidental exposures, usually through drinking beverages contaminated with methanol. Other studies reported the results from different treatments (fomepizole, hemodialysis, folic acid and ethanol) and the clinical evolution, depending on the time when the treatment began.

**Table 1.** Databases, search strategies and results from systematic search in the electronic databases

Database	Search strategy	Results
PubMed	Methanol AND suicide, attempted	6 clinical trials 15 case reports 3 reviews
Embase	Methanol, poisoning AND suicide, attempted	2 case reports
Cochrane library	Methanol , qualifiers: diagnosis, toxicity, poisoning	1 case-control study 2 clinical trials 1 case report
Lilacs (Literatura Latino-americana e do Caribe em Ciências da Saúde)	Methanol , qualifiers: poisoning, toxicity	4 case reports 3 reviews 1 clinical trial 1 epidemiological study 1 environmental study

MeSH = Medical Subject Headings; DeCS = Descritores em Ciências da Sáude.

## CONCLUSION

The present case could be classified as one of potentially severe methanol poisoning, according to the amount and concentration of methanol ingested, and the blood methanol concentrations 4 h after ingestion. The good outcome was probably attributable to the early hospital admission and the early antidotal therapy together with hemodialysis, which started 10 and 19 h after ingestion, respectively.
